# Mental health status and related influencing factors in patients with COVID-19

**DOI:** 10.1186/s40359-023-01254-8

**Published:** 2023-08-07

**Authors:** Ying He, Lei Huang, Jian Chen, Ling Long, Ling Zhang, Xiao Hui, Qingling Zhang, Muzhen Guan, Yuanjun Xie, Jianguo Sun

**Affiliations:** 1 Department of Medical Psychology, Neurology Medical Center, The Second Affiliated Hospital, Army Medical University, Chongqing, China; 2 Cancer Institute, The Second Affiliated Hospital, Army Medical University, Chongqing, China; 3 Department of Mental Health, Xi’an Medical College, Xi’an, China; 4 Department of Military Medical Psychology, Fourth Military Medical University, Xi’an, China

**Keywords:** Mental health status, Influencing factors, Patients, COVID-19

## Abstract

**Background:**

The outbreak of the Corona Virus Disease 2019 (COVID-2019) has resulted in a significant number of diagnosed patients requiring treatment in designated hospitals. However, limited evidence exists regarding the prevalence of mental health problems and associated psychological factors in COVID-19 patients.

**Objectives:**

This study investigated the prevalence rates of anxiety, depression, and insomnia among COVID-19 patients, as well as explored the associations between these mental health problems and psychological factors.

**Methods:**

A cross-sectional study was conducted among 387 COVID-19 patients in a designated shelter hospital. Online measures were used to assess anxiety, depression, insomnia, social support, coping styles, and emotional regulation. Data were analyzed to determine the prevalence rates of mental health problems and examine the associations between these problems and the psychological factors.

**Results:**

The results revealed high prevalence rates of anxiety (21.80%), depression (49.0%), and insomnia (63.70%) among COVID-19 patients. Objective social support scores and positive coping style scores were significantly associated with lower levels of anxiety, depression, and insomnia, respectively. Conversely, high negative coping style scores, higher education level, and self-perceived illness severity, were significantly related to higher levels of anxiety, depression, or insomnia symptoms. Emotional regulation scores did not show a significant association with any of the examined mental health problems.

**Conclusions:**

These findings have implications for guiding psychological interventions tailored to COVID-19 patients in future outbreaks. By targeting social support and promoting adaptive coping strategies, psychological interventions can address the psychological distress experienced by COVID-19 patients.

## Background

The outbreak of coronavirus disease 2019 ( COVID-19) originated in China and rapidly spread to numerous countries, leading to a global pandemic [1]. The escalating global morbidity and mortality rates associated with COVID-19 have raised significant public health concerns [2], affecting more than 281 million individuals worldwide [3]. The immediate psychological impact of the COVID-19 outbreak has been extensively studied in China. A survey assessed the initial psychological response of the general public after two weeks into the COVID-19 outbreak in China [4]. The findings indicated that 16.5% of patients reported moderate to severe depressive symptoms, 28.8% reported moderate to severe anxiety symptoms, and 8.1% reported moderate to severe stress levels. The prevalence of psychological problems was higher among individuals affected by the disease compared to the general population [5]. Similar findings have been observed in other countries as well [6].

Previous studies have primarily focused on the mental health implicates of COVID-19 among the general population, healthcare workers, and individuals in quarantine [7, 8]. However, the psychological impacts on individuals infected with the virus should also be considered. Currently, there is limited and unclear evidence regarding the prevalence of mental disorders in patients with COVID-19. A recent cross-sectional study examined the psychological problems of patients with COVID-19 who were treated in designated hospitals in Wuhan, China [9]. The study revealed a higher prevalence of anxiety, depression, and sleep disturbance among these patients. Similarly, Yadav and colleagues investigated the psychological distress experienced by patients with COVID-19 at a tertiary care center in North India [10]. Their findings indicated that 27% of patients experienced depression, 67% reported anxiety, and 62% had sleep disorders. Moreover, observational studies [11] and meta-analysis studies [12] have consistently demonstrated that patients with COVID-19 are more susceptible to anxiety, depression, and sleep problems due to the highly infectious nature of the disease. These findings suggested that individuals with COVID-19 are at a heightened risk of developing mental health issues.

Several factors have been identified to impact individual mental health, including social support, coping styles, and emotion regulation strategies. Social support plays a crucial role in reducing psychological distress [13]. Adequate social support has been shown to alleviate depression, and anxiety symptoms [14] and positively influence on sleep quality [15]. Conversely, individuals lacking access to social support tend to experience higher levels of anxiety and depression symptoms [16]. Coping styles are also associated with mental health outcomes during the COVID-19 pandemic [17]. Positive or problem-focused coping styles have been found to alleviate symptoms of depression, insomnia, and post-traumatic stress symptoms [18], while negative or emotion-focused coping styles may exacerbate mental health symptoms [19]. Additionally, emotion regulation is strongly linked to overall well-being [20]. Adaptive emotion regulation strategies enable individuals to cope with environmental stressors [21] and have been shown to reduce COVID-19-related anxiety [22], depression [23], and sleep disturbance [24].

While previous studies have extensively examined the relationship between elements such as social support, coping style, and emotion regulation, and their potential influence on the severity of anxiety, depression, and insomnia in the general public [25–27] and frontline healthcare workers [28–30] during the COVID-19 pandemic, limited attention has been given to these associations in patients with COVID-19. Understanding the psychological and social factors that can help protect against psychological distress in patients with COVID-19 is crucial. Therefore, the aim of this cross-sectional study was to investigate the mental health status of patients with COVID-19 and explore the potential factors, including social support, coping styles, and emotion regulation, that are associated with mental health status.

## Methods

### Participants

This cross-section investigation was conducted using an anonymous online questionnaire from April 9, 2022, to May 10, 2022. The participants of the study were recruited from inpatients with COVID-19 in a designated shelter hospital in Shanghai, China. Participants completed online questionnaire prior to their discharge. The inclusion criteria for the patients were as follows: (1) a stable state of consciousness, (2) ability to understand and complete the questionnaire, and (3) being easily approachable during the investigation. Patients were diagnosed based on the guideline for COVID-19 (eighth edition) issued by the National Health Commission of China [31]. Confirmation of the diagnosis was done through chest CT scanning or nasopharyngeal swab testing with real-time reverse transcription-polymerase chain reaction (RT-PCR). Exclusion criteria were included: (1) previously diagnosed serious mental disorders such as schizophrenia and bipolar disorder, (2) current oral medication for a chronic condition that causes side effects related to mental health issues such as anxiety and depression, and (3) refusal to provide consent to participation. A total of 407 inpatients met the specified criteria and were included in the study.

### Procedure

This study was conducted by doctors working at the hospital and was approved by the Medical Ethics Committees of the Second Affiliated Hospital of Army Medical University (No.2022-332-01). Participants were provided information about the purpose of the study and assured that their data would remain confidential. They were also asked to review and sign an informed consent form indicating their voluntary participation in the study. Participants were then asked to complete a series of questionnaires that included socio-demographic information, clinical characteristics, and mental health status measurements.  

### Measures

#### Demographic and clinical characteristics

The patients in this study completed the online questionnaires by scanning the quick response code with their mobile phones. The self-designed questionnaires to collect data on demographic and clinical characteristics. The detailed information collected included gender, age, marital status, education level, employment status, oral medication, hospitalization period, and self-perceived illness severity.

#### Mental health status

Symptoms of anxiety were evaluated using the Generalized Anxiety Disorder-7 (GAD-7) scale, a self-administered screening tool devised for the detection of potential anxiety. The GAD-7 scoring system ranges from 0 to 21, categorizing anxiety severity into four levels: no anxiety (0–5), mild anxiety (6–10), moderate anxiety (10–15), and severe anxiety (16–21) [32]. Symptoms of depression were ascertained by the Patient Health Questionnaire-9 (PHQ-9) scale, a self-reporting diagnostic tool for depression. The PHQ-9 scoring framework spans from 0 to 27, designating depression severity classified into five categories: no depression (0–4), mild depression (5–9), moderate depression (10–14), moderately severe depression (15–19), and severe depression (20–27) [33]. Sleep problems were evaluated by Athens Insomnia Scale (AIS), a self-reported instrument specifically designed to determine the insomnia severity. Comprising 8 items rated from 0 to 3, the AIS considers a cumulative score exceeding 8 as indicative of insomnia [34]. These three scales have demonstrated strong reliability and validity in previous studies [35, 36], and in this study, their Cronbach’s alpha values were 0.949, 0.925, and 0.9, respectively.

In addition, this study evaluated social support using the Social Support Rating Scale (SSRS). This ten-item scale gauges three facets of social support: objective support, subjective support, and support utilization [37]. It has exhibited robust reliability and reliability [38], with Cronbach’s alpha values in this study being 0.893, 0.825, and 0.896, respectively, for the three scales. Coping styles were scrutinized via the Simplified Coping Style Questionnaire (SCSQ) [39]. This 20-item questionnaire assesses two coping styles: positive coping style and negative coping style, and has previously demonstrated strong reliability and validity [40]. In this study, the Cronbach’s alpah values were 0.925 and 0.810, respectively. Emotion regulation strategies were assessed using the Emotion Regulation Questionnaire (ERQ) which comprises 10 items to measure two particular strategies: cognitive reappraisal and expressive suppression [41]. This tool has shown good psychometric properties [42], and in this study, the two subscales’ Cronbach’s alpah values were 0.919 and 0.876, respectively.

### Statistical analyses

All statistical analyses were conducted using IBM SPSS version 26 (IBM Corporation, Armonk, NY, USA). Categorial data were presented as frequencies and percentages, while continuous data were presented as mean ± standard deviation (SD). Multiple linear regression analysis was performed to identify the independent factors associated with anxiety, depression, and insomnia, including social support, coping styles, emotional regulation, and demographic variables. The enter method was used for linear regression. For all statistical analyses, a threshold of *p* < 0.01 was established to denote statistical significance.

## Results

### Demographic and clinical characteristics

A total of 407 patients with COVID-19 participated were initially enrolled in this study, and 387 of them completed the survey, resulting in an effective response rate of 95%. Table 1 presents the demographic and clinical characteristics of the participants. The mean age was 41 years (SD = 14 years). More than half the patients were male (53.20%), married (69.80%), and employed (75.50%).

**Table 1:** General characteristics of participants (N = 387)

Variable	Mean ± SD / n (%)
**Age** (year)	41 ± 14
**Sex**	
Male	206 (53.20)
Female	181 (46.80)
**Education level**	
Primary school	56 (14.50)
Junior school	122 (31.50)
High school	110 (28.40)
College and above	99 (25.60)
**Marital status**	
Single	101 (26.10)
Married	270 (69.80)
Divorced	16 (4.10)
**Employment status**	
Employed	292 (75.50)
Unemployed	61 (15.80)
Retirement	34 (8.80)
**Oral medication**	
Chinese medicine prescription	101 (26.10)
Kangbingdu Granules	92 (23.80)
Lianhua Qingwen	100 (25.80)
Cough medicine	94 (24.30)
**Hospital stays**	
≤ 7 days	287 (74.20)
8–14 days	100 (25.80)
**Self-perceived illness severity**	
Normal	145 (37.50)
Severity	129 (33.30)
Very severity	113 (29.20)

### Assessment of mental health status

**Fig. 1:**
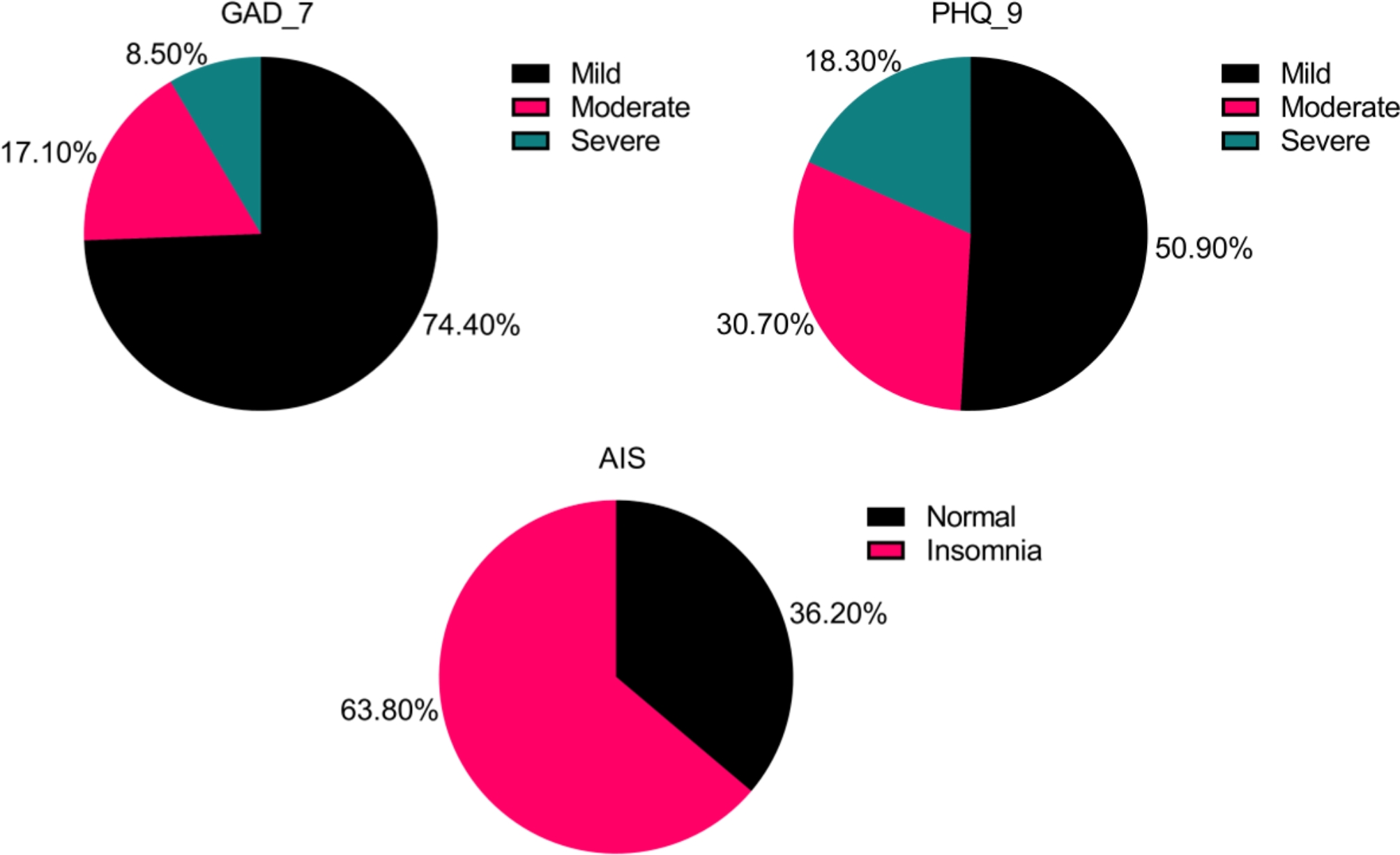
The distribution of levels of severity on mental health status among patients with Corona Virus Disease 2019. GAD, generalized anxiety disorder; PHQ, patient health questionnaire; AIS, Athens Insomnia Scale

The mean scores of GAD_7, PHQ_9, and AIS were 9.56 (SD = 4.48), 12.61 (SD = 5.30), and 12.02 (SD = 4.91), respectively. Figure 1 presents the distribution of participants across the three levels of severity for anxiety, depression, and insomnia. According to the PHQ-9, 25.60% of patients had moderate to severe depression, with 33 (8.50%) falling into the severe category. Using the GAD-7,190 patients (49.0%) were classified as having moderate to severe anxiety. For the AIS, 36.30% of patients reported normal sleep patterns, while 63.80% experienced insomnia.

### Factors associated with mental health status

**Fig. 2:**
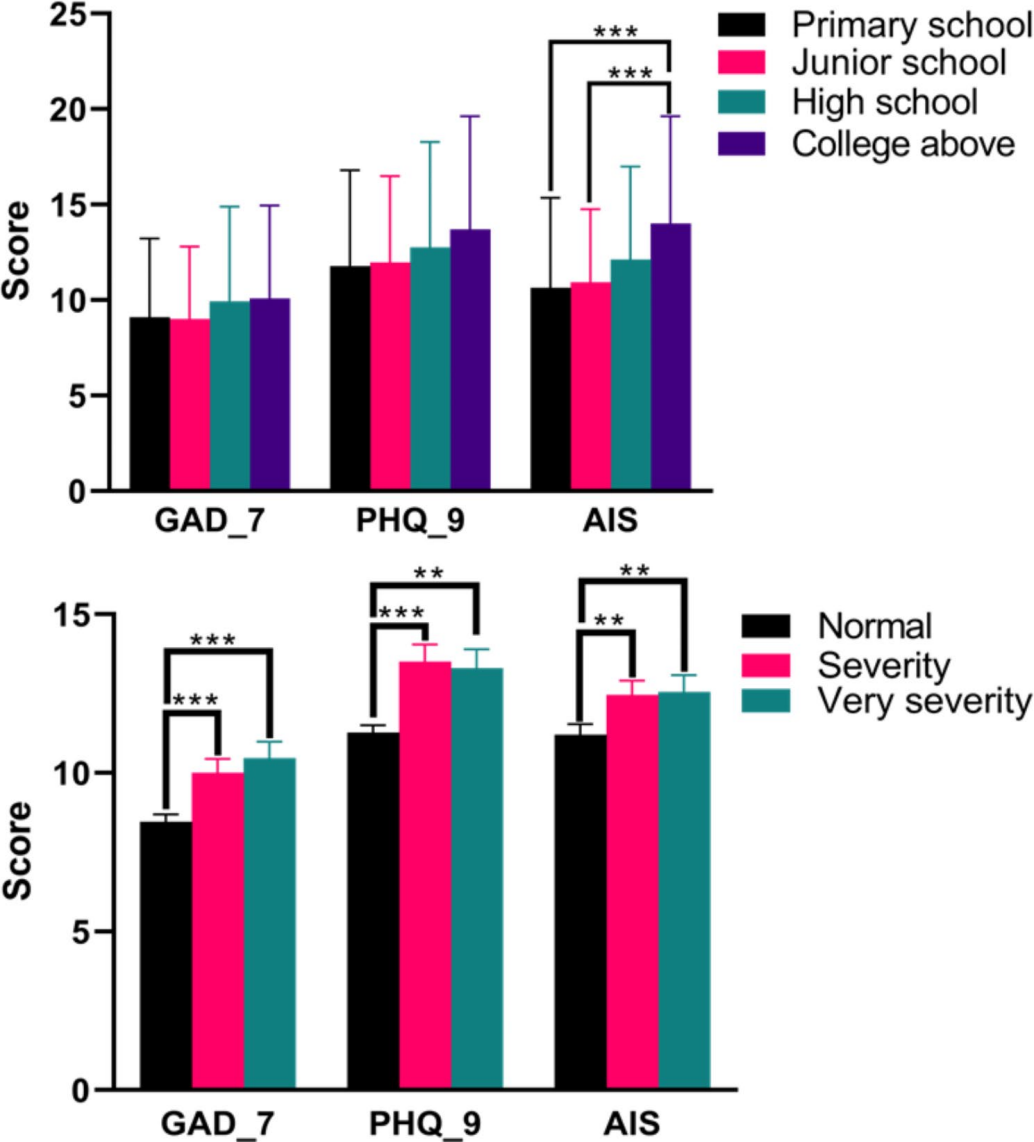
The univariate analyses on the mental health status of patients with Corona Virus Disease 2019. GAD, generalized anxiety disorder; PHQ, patient health questionnaire; AIS, Athens Insomnia Scale. ^**^p < 0.01, ^***^p < 0.001

Figure 2 displays the outcomes of the univariate analyses conducted on the patient sample. The results indicated that patients possessing higher levels of education, such as a college degree or above, reported poorer sleep quality compared to those with only primary and junior school education (t = 3.774, *p < 0.01*; t = 4.799, *p < 0.01*). Furthermore, patients perceiving their disease as severe or very severe manifested elevated levels of anxiety (t = 3.271, *p < 0.01*; t = 3.858, *p < 0.01*) and depression (t = 3.981, *p < 0.01*; t = 3.429, *p < 0.01*), in contrast to those with a normal disease perception. Similarly, those reporting a severe to very severe disease perception experienced more pronounced sleep disturbances than individuals perceiving their disease as normal (t = 2.267, *p* = 0.008; t = 2.350, *p* = 0.007).

Table 2 illustrates the results from the multiple linear regression models pertaining to GAD_7, PHQ_9, and AIS scores. Notably, certain variables consistently exhibited significance within the regression model. Specifically, both objective support and positive coping style were inversely related to GAD_7 scores (Beta = -0.159, t = 2.722,  *p* = 0.007; Beta = -0.258, t = 3.759, *p < 0.01* ), PHQ_9 scores (Beta = -0.170, t = 2.940, *p* = 0.003; Beta = -0.303, t = 4.456, *p < 0.01*), and AIS scores (Beta = -0.215, t = 3.176, *p* = 0.002). Contrastingly, a negative coping strategy was positively associated with GAD_7 scores (Beta = 0.152, t = 2.709, *p* = 0.007) and PHQ_9 scores (Beta = 0.184, t = 3.303, *p* = 0.001). Additionally, self-perceived disease severity and education level emerged as substantial predictors of anxiety symptoms (Beta = 0.181, t = 3.630, *p < 0.01*; Beta = 0.191, t = 3.516, *p < 0.01*), depression symptoms (Beta = 0.161, t = 3.200, *p* = 0.001; Beta = 0.188, t = 3.492, *p* = 0.001), and insomnia (Beta = 0.114, t = 2.240, *p* = 0.026) among the patients. However, emotion regulation strategies did not demonstrate associations with GAD_7 scores, PHQ_9 scores, or AIS scores in these models.

**Table 2:** Predictors of GAD_7, PHQ_9, and AIS among COVID-19 patients

		GAD_7				PHQ_9				AIS		
Predictor		Beta	t	*p*		Beta	t	*p*		Beta	t	*p*
**SSRS**												
Objective support		-0.159	2.722	0.007		-0.170	2.940	0.003		-0.133	2.311	0.021
Subjective support		0.056	0.828	0.408		0.001	0.013	0.990		-0.030	0.446	0.656
Support utilization		0.004	0.060	0.952		0.028	0.427	0.670		0.076	1.181	0.238
**SCSQ**												
Positive coping		-0.258	3.759	0.000		-0.303	4.456	0.000		-0.215	3.176	0.002
Negative coping		0.152	2.709	0.007		0.184	3.303	0.001		0.062	1.120	0.263
**ERQ**												
Cognitive appraisal		0.097	1.486	0.138		0.102	1.585	0.114		0.090	1.391	0.165
Expressive suppression		-0.046	0.744	0.458		-0.082	1.336	0.183		-0.049	0.803	0.422
**Sex**		0.093	1.824	0.069		0.067	1.331	0.184		0.094	1.874	0.062
**Education level**		0.191	3.516	0.000		0.188	3.492	0.001		0.299	5.595	0.000
**Marital status**		0.140	2.307	0.022		0.067	1.115	0.266		0.066	1.112	0.267
**Employment status**		-0.066	1.236	0.217		-0.041	0.770	0.442		-0.043	0.810	0.418
**Oral medication**		-0.066	1.320	0.188		-0.076	1.507	0.133		-0.032	0.623	0.533
**Hospital stays**		0.098	1.954	0.051		0.049	0.977	0.329		0.054	1.057	0.291
**Illness severity**		0.181	3.630	0.000		0.161	3.200	0.001		0.114	2.240	0.026

Abbreviations: COVID-19, Coronavirus disease 2019; GAD, Generalized Anxiety Disorder; PHQ, Patient Health Questionnaire; AIS, Athens Insomnia Scale; SSRS, Social Support Rating Scale; SCSQ, Simplified Coping Style Questionnaire; ERQ, Emotion Regulation Questionnaire.

## Discussion

The present study investigated the mental health status and identified related factors in COVID-19 patients. The findings revealed that anxiety and depression were the most prevalent psychological distress, accompanied by a high prevalence of insomnia. Factors that negatively affected the mental health of patients included negative coping style, higher levels of education, and self-perceived illness severity. On the other hand, objective support and positive coping style were found to be protective factors against psychological distress. These findings contribute to our understanding of the factors associated with psychological stress in COVID-19 patients and provide valuable insights into the mental health status of patients during quarantine periods.

The present study revealed significant psychological distress among COVID-19 patients, with high morbidity of anxiety (25.6%), depression (49.0%), and insomnia (63.8%). These findings align with previous survey [7] and research conducted during other epidemics [43], such as severe acute respiratory syndrome (SARS), Ebola virus, and middle east respiratory syndrome (MERS), where patients also experienced elevated levels of anxiety, depression, and sleep problems [44–46]. The observed correlation between patients’ self-perception of COVID-19 severity and increased symptoms of anxiety, depression, and sleep disturbances underscores the significant psychological burden associated with the pandemic. The heightened anxiety and depression symptoms among patients perceiving their COVID-19 disease severity as high might be primarily attributable to the elements of fear and uncertainty. Fear regarding the potential outcomes of a severe illness, such as enduring disability or mortality, can evoke considerable emotional distress [47]. Concurrently, uncertainty surrounding the progression and prognosis of COVID-19, especially in severe cases, may incite anticipatory anxiety and concern, leading to depressive symptoms [48]. The association between perceived disease severity and sleep disruptions can be interpreted within the framework of stress-induced insomnia. It is well documented that stress, especially chronic or intense, can lead to sleep disturbances or insomnia [49]. Inherently, perceiving one’s disease as severe can be a source of significant stress, and the cognitive arousal it evokes – encompassing intrusive thoughts and excessive worrying – can hinder sleep onset or maintenance. Such cognitive arousal tends to peak at bedtime, a period marked by fewer distractions and increased solitude, thereby leading to deteriorated sleep quality and insomnia [50].

Intriguingly, this study revealed that COVID-19 patients with higher education levels (college or above) experienced more severe sleep disturbances than those with primary or junior school education. This may initially seem counterintuitive as a higher education level is generally associated with improved health outcomes [51]. However, when interpreted in the context of a pandemic, the relationship between educational attainment and sleep disturbances becomes multifaceted. A plausible explanation could be the degree of information exposure and consumption. Individuals with higher educational qualifications are likely to follow updates about COVID-19 more closely and comprehend the evolving scientific discourse surrounding the virus, which often contains distressing information. Exposure to such pandemic-related news has been linked to sleep disturbances [52]. Cognitive processes might also contribute to this phenomenon. Higher educational attainment is often associated with increased cognitive rumination, characterized by repetitive thinking about one’s problems and associated emotions. In the context of negative or stressful situations, such as a pandemic, rumination can intensify emotional responses, contributing to sleep difficulties [53]. Nonetheless, these interpretations are preliminary, and this unexpected finding merits further research to uncover the underlying mechanisms.

It is indeed intriguing that objective support, rather than subjective support or support utilization, was found to significantly alleviate the level of anxiety, depression, and insomnia in patients. Previous studies have consistently demonstrated the positive effects of social support on improving psychological well-being. Social support can enhance the individuals’ sense of self-protection [54] and effectively mitigate the negative impact of stressful events on the mental health. During the COVID-19 pandemic, an enforced lockdown policies and restrictions limited the availability of subjective support from family and friends. Therefore, receiving tangible and concrete support (e.g., objective support) from the government agencies, non-government organizations, and local community [55], or through online platforms and social media [56] became crucial in helping patients cope with stress and improve their mental health. Particularly, support from social media and online communication platforms can provide access to health information [57] and facilitate interactions with others in the similar condition [58], which plays an important role in how patients respond to COVID-19. The findings of this study suggest that patients who reported higher levels of objective social support were less likely to experience symptoms of anxiety, depression, and insomnia. This highlights the significance of actual social support as a protective factor for the mental health of patients with COVID-19 in isolated environments. By receiving visible and tangible support, patients can feel more supported and connected, which can buffer the psychological impact of the pandemic and promote better mental well-being.

Indeed, coping styles play an important role in mental health outcomes [59]. Positive coping style, characterized by problem-focused coping strategies such as seeking advice from others or finding multiple solutions to problem, has been associated with better mental health outcomes [60]. On the other hand, negative coping style, which emphasize helplessness and passivity, tends to be associated with increased feelings of depression and anxiety [61]. In line with the previous studies, the current study found that a higher found positive coping style was associated with fewer symptoms of anxiety, depression, and insomnia, while negative coping style showed the opposite pattern. These findings suggested that adopting a positive coping style, focusing on active problem-solving and seeking support, serves was a protective factor for the mental health of patients. Additionally, the study identified higher education levels, being divorced, and self-perceived illness severity as risk factors for anxiety, depression, and insomnia. As mentioned above, individuals with higher education levels or who are divorced and self-perceived illness severity are particularly vulnerable to developing mental health problems during the COVID-19 pandemic [62–64], This suggests that patients with higher education levels or those who are divorced and perceive their illness as more severity may experience exacerbated mental health difficulties in the context of the pandemic.

Cognitive reappraisal and expressive suppression are two commonly unitized emotion regulation methods. Cognitive reappraisal has been shown to enhance positive emotions and prevent distress [65]. Conversely, expressive suppression can intensify negative emotions and increase distress [66]. Surprisingly, the current survey did not find an association between emotion regulation and the mental health status of patients with COVID-19. This finding may be attributed to the unique circumstances faced by COVID-19 patients, such as enforced quarantine and the perceived threat to life. Under these circumstances, strategies like changing the meaning of a situation and inhibiting emotional expression may be ineffective. The high prevalence of anxiety and depression among patients provides indirect evidence supporting this notion.

There are a few limitations to our study that should be acknowledged. Firstly, the study design was cross-sectional, which limits our ability to establish causal relationships between mental health issues and the related factors. Longitudinal studies would provide a stronger basis for understanding the temporal dynamics and determining causal links between variables. Secondly, our study was conducted exclusively among COVID-19 patients treated in designated hospitals. Therefore, caution should be exercised when generalizing the results to the general population or individuals with different demographic or clinical characteristics. Future research should consider including a more diverse and representative sample to enhance the external validity of the findings. Thirdly, our study lacked the collection of socioeconomic and biochemical indices, such as household income and inflammatory markers. Future research is needed to collect these additional measures, which provide a more comprehensive understanding of mental health-related factors.

## Conclusions

The present study conducted a cross-section analysis of the mental status and influencing factors in patients with COVID-19. The finding revealed that COVID-19 patients experience psychological distress and sleep disturbances, and identified critical factors including objective support and coping styles as associated factors. These findings provide valuable insights into the mental health status of COVID-19 patients and highlight potential areas for psychological intervention in future outbreaks. By understanding the specific factors that contribute to psychological distress, healthcare professionals can develop targeted interventions to support the mental well-being of patients in similar circumstances.

## Data Availability

All data is freely available upon request from the corresponding author. Data are not publicly available due to privacy and ethical constraints.
